# Dietary Behaviors That Place Young Adults at Risk for Future Osteoporosis

**DOI:** 10.3390/nu12061800

**Published:** 2020-06-17

**Authors:** Alyse Davies, Anna Rangan, Margaret Allman-Farinelli

**Affiliations:** Nutrition and Dietetics Group, School of Life and Environmental Science, Charles Perkins Centre, The University of Sydney, Sydney, NSW 2006, Australia; anna.rangan@sydney.edu.au (A.R.); margaret.allman-farinelli@sydney.edu.au (M.A.-F.)

**Keywords:** peak bone mass, bone mineral density, food groups, calcium, sodium, alcohol

## Abstract

Dietary behaviors during adolescence and emerging adulthood have important consequences for peak bone mass (PBM) attainment. This study aimed to examine dietary factors that are either beneficial or detrimental to bone health and determine the major sources of calcium in the diets of a sample of young adults. A cross-sectional survey was conducted among 189 Australians aged 18–30 years. Three-day dietary intakes were collected using consecutive 24 h recall interviews. Daily totals for energy and nutrients and serves for food groups were computed. The proportion contribution of calcium (mg) from different food groups as well as calcium (mg) per portion and per 100 g were calculated. Females and males failed to meet the recommendations for dairy (91%, 82%), fruit (89%, 94%) and vegetables (74%, 86%). Eighty percent were above the recommended daily intake range for sodium. For calcium, 53% of females and 48% of males had intakes below the estimated average requirement (EAR). Milk products and dishes made the highest mean calcium contribution per portion (mg) mean standard deviation (SD), 204 mg (212) and accounted for 30% of calcium intake in females and 35% in males. As young adulthood is the final chance for dietary manipulation before PBM is achieved, these dietary risk factors should be addressed.

## 1. Introduction

Osteoporosis is a skeletal condition that predisposes a person to fractures, categorized by a reduction in bone mineral density (BMD) and microarchitecture deterioration [1]. The World Health Organization (WHO) established diagnostic criteria for osteoporosis and a clinical diagnosis is made when BMD is 2.5 standard deviations (SD) or more below that of a young adult reference population [2]. Osteoporosis is a debilitating disease that has become a major public health concern affecting 200 million people worldwide [3]. It has been estimated that in Australia in 2017, the total direct cost of osteoporosis was Australian Dollars AUD 3.44 billion and this will only increase with the aging population [4]. As osteoporosis presents as a silent disease, meaning that there are no clinical manifestations until a fracture occurs, the condition is often under-diagnosed [5].

There is extensive evidence suggesting that early life experiences can predict the risk of acquiring osteoporosis in the future [6]. While genetic factors play a significant role in determining whether an individual is at an increased risk of acquiring the disease, 20–40% of adult peak bone mass (PBM) is influenced by dietary and lifestyle factors, which are modifiable and can help protect against the disease [7]. Bone mineral accrual is rapid during childhood and adolescence as the skeleton is growing, modelling, and remodelling through the process of bone formation and resorption [6]. Peak bone mass is described as the total amount BMD accrued by the end of skeletal maturation which is reached early in the third decade of life [6]. Therefore, young adulthood may be seen as the last chance to improve dietary and lifestyle factors to optimize PBM.

Diet quality is often compromised during the transition between adolescence and young adulthood [8]. Young adults often leave the family home and begin to take responsibility for their own meals. The increase in autonomy and independence is one that may result in a higher intake of meals prepared outside the home and a lower consumption of foods from the recommended food groups [9]. Dairy products such as milk, yoghurt and cheese contain high amounts of calcium and the Australian Guide to Healthy Eating (AGHE) recommends young adults have two and a half serves a day [10]. Calcium is an essential nutrient required for PBM growth during adolescence and into adulthood [7]. There is accumulating evidence suggesting fruit and vegetables have protective effects on bone health through mediation of the body’s acid-base balance [11]. Fruit and vegetables contain alkaline-forming properties which act as a buffer for acidic-forming foods. A more alkaline environment has been shown to reduce urinary calcium excretion, while a more acidic environment may cause a fall in the pH, stimulating bone resorption [12].

The major dietary factors that have been shown to be detrimental to bone health include an excessive intake of alcohol, sodium and caffeine [13]. Alcohol has deleterious effects on bone when consumed in high amounts, damaging bone tissue because it interferes with bone growth and remodelling [14]. Both high intakes of sodium [15] and caffeine [16] can increase calcium excretion and a higher calcium excretion is associated with a lower BMD and subsequent risk of fracture. An adequate daily intake of protein is essential for bone health as bones continually undergo modelling and remodelling but intakes that are either insufficient or in excess can be detrimental [13].

The objective of this study was to examine dietary behaviors that are either beneficial or detrimental to bone health and determine the major sources of calcium in the diet of young adults. Such information might be used when designing interventions to improve dietary behaviors to optimize PBM.

## 2. Materials and Methods

### 2.1. Sample

The sample population of this study comprises a subset of a larger cross-sectional study (MY Meals) who consented to the collection of dietary data via three 24 h dietary recalls [17]. The eligibility criteria in brief; were those aged 18 to 30 years, English-speaking, purchased and consumed at least one meal, snack or drink outside of the home setting each week and owned a working smartphone. Participants were excluded if they had ever been diagnosed with an eating disorder, were pregnant/breastfeeding or did not complete the three study days [17]. Participants were recruited across New South Wales (NSW), Australia using a range of recruitment methods including personalized letters posted to one thousand participants targeting rural and low socio-economic areas across NSW using the Australian Electoral Commission roll; paid social media advertising (Facebook and Instagram); Cancer Council NSW fundraising events and posters and electronic media at the University. Participants received an AUD 100 gift voucher as a reimbursement for their time after all study requirements were completed. Ethics approval was obtained by the Human Research Ethics Committee (project 2016/546).

### 2.2. Data Collection, Demographics Characteristics and Anthropometry

Potential participants completed an online screening and demographics questionnaire. Dietary data were collected using three consecutive 24 h recalls of all food and beverages administered by dietitians using the standardized automated ASA24 Australia, which uses an Australian database of foods [18]. This multiple pass computerized method prompts for additional information on food form, preparation methods, portion sizes and commonly forgotten items. Demographics in the analysis included; gender (male or female); age (18 to 24 and 25 to 30 years); socio-economic status (SES) assessed using residential postcode to assign the index of relative socio-economic advantage and disadvantage centile employed within Australia (high, low) [19]; educational attainment (secondary school or less, trade qualification, diploma or university degree). Secondary school or less includes those who have either left school with no further study or those with high school as their highest attainment but currently studying. Country of birth (born in Australia, other country or prefer not to say) and geographic location (Sydney, outer Sydney or regional) which was assessed by the Accessibility Remoteness Index of Australia [20]. At the conclusion of the three 24 h recalls the participants completed an online demographic questionnaire which included questions on self-reported height and weight to calculate Body Mass Index (BMI) and a sample were measured by research staff to validate this measure [21].

### 2.3. Nutrients and Nutrient Densities

Daily totals for energy, dietary fibre and % energy for macronutrients were computed, then means (SD) were calculated for the three study days for protein, total and saturated fat, carbohydrate, alcohol and fibre. Daily totals for calcium, sodium, caffeine were computed, then median interquartile range (IQR) and nutrient densities (mg/1000 kJ) were calculated for the three study days. Vitamin D could not be estimated as they are not in the Australian food composition database. Supplements were excluded in analysis.

### 2.4. Serves for Selected Food Groups

The food groups used in this analysis include fruit; vegetables and legumes/beans; and milk, yoghurt cheese and/or alternatives. For simplicity purposes, these categories will be referred to as fruit, vegetables and dairy and/or alternatives throughout. To assess the number of serves consumed within these food groups, all individually recorded food items as well as foods that were part of a mixed dish were included. Detailed measures of all individual food components were obtained by disaggregating all mixed dishes using the AUSNUT 2011–2013 recipe file. Serves for fruit, vegetables and dairy and/or alternatives were summed and averaged over the three days of recording. Food group serves were calculated using standard serves outlined by the AGHE [10]. A serve of fruit was calculated as 150 g and 125 mL for fruit juice; a serve of vegetables was calculated as 75 g and 125 mL for vegetable juice; a serve of dairy and/or alternatives was calculated as 250 mL for milk and milk alternatives, 40 g of hard cheese, 120 g of ricotta cheese and 200 g of yoghurt. A standard drink of alcohol is any drink containing 10 g of alcohol.

### 2.5. Assessment of Calcium Contribution by Food Categories

Foods were classified according to the Australian National Health Survey (NHS): User’s Guide, 2011–2013 Data item list [22], which was developed by Food Standards Australia New Zealand along with the nutrient database. The system was designed to capture food reported by the participants in the NHS, particularly mixed dishes. Thirteen major groups (broadest level), sub-major and minor food groups that contributed 1.5% or more calcium (mg) for either gender were included. The selected major food groups were; milk products and dishes; cereal-based products and dishes; non-alcoholic beverages (includes items prepared with or without milk); cereals and cereal products; vegetable products and dishes; dairy and meat substitutes; meat, poultry and game products and dishes; confectionary and cereal/nut/fruit/seed bars; chocolate and chocolate based confectionary; fruit products and dishes; fish and seafood products and dishes; egg products and dishes; special dietary foods (includes items prepared with or without milk such as meal replacement or sports/protein powders or beverages). For categorization purposes, foods were grouped based on the major component even for mixed dishes. For example, cereal-based products and dishes are mixed dishes with cereal as the major component, but all other non-cereal foods are also included i.e., all components of a pizza, including the base, cheese and toppings were included in the group cereal-based products and dishes. The average portion size (g) (i.e., the amount of food consumed per eating occasion) for each major, sub-major or minor food category was calculated as well as calcium as mg, per portion and mg, per 100 g with the mean (SD) reported. The percentage of calcium sourced from a particular food group was calculated across the sample as sum of calcium (mg) from food group divided by sum of calcium (mg) from all food groups for both genders.

### 2.6. Statistical Analysis

The Schofield equation was used to calculate basal metabolic rate (BMR) where BMR = (62 × weight) + 2036 for females and (63 × weight) + 2896 for males. The Goldberg cut-off method of (energy intake: basal metabolic rate, EI:BMR) was used to identify potential low energy reporters (<1.00 EI:BMR, *n* = 28) and over-reporters (>2.4 EI:BMR, *n* = 6) from the average energy intake over the three days [23]. Independent *t*-tests were conducted on normally distributed data to compare the mean nutrient and food intakes between genders. Mann-Whitney U test was used for non-parametric data including the comparison of food serves and SES. Statistical analyses were performed using IBM SPSS Statistics, version 24 for windows with *p* < 0.05 deemed statistically significant.

## 3. Results

### 3.1. Characteristics

A total of 216 participants were recruited to this sub-study. Five participants withdrew for either personal or employment reasons, twenty did not complete all study days and two did not meet the eligibility criteria, leaving a sample of 189 young adults. Table 1 summarises the characteristics of the participants. The sample had slightly fewer males (46%) than the Australian population proportion (49%) [24] and less living in major cities (66%) than the population proportion (72%) [25]. Among participants 65% were from higher SES and 65% had tertiary education (trade, diploma or university degree), higher than the 56% reported by the Australian Bureau of Statistics (ABS) [26]. More individuals were in the normal weight range 63% compared to the 2017–2018 NHS, where 50% of 18–24 years old and 41% of 25–34 years old were normal weight [27].

### 3.2. Comparing Energy and Nutrient Densities Between Genders and Socio-Economic Status

Energy adjustment was used to control for measurement error by reporting the proportion of energy (% energy) for macronutrients and nutrient densities (mg/1000 kJ) for micronutrients. A sensitivity analysis was conducted removing the low energy reporters (*n* = 28). This analysis did not change the results for calcium density (*p* = 0.22), therefore the total sample (*n* = 189) was included in the analysis.

There was no significant difference (*p* = 0.16) in the median (IQR) for calcium (mg/day) between females 801 mg (639–1017) and males 871 mg (680–1213) nor for calcium densities (*p* = 0.52) between females 90 mg (76–115) and males 90 mg (67–112). Fifty three percent of females and 48% of males had median intakes below the estimated average requirement (EAR) (840 mg per day). There was no significant difference (*p* = 0.51) in the median (IQR) for calcium densities between high 89 mg (70–115) and low 93 mg (74–114) SES groups.

A significant difference (*p* < 0.001) in the median (IQR) for sodium (mg/day) was observed, with males having a higher intake 3091 mg (2443–3810) compared to females 2643 mg (1888–3271), but when reporting nutrient densities, no significant difference (*p* = 0.48) was observed. For sodium, 80% were above the WHO recommended daily intake of 2000 mg/day, which is equivalent to 5 g of salt per day. There was no significant difference (*p* = 0.65) in the median (IQR) for sodium densities between high 294 mg (233–348) and low 295 mg (245–360) SES groups.

There was a significant difference found between genders for caffeine (*p* = 0.01) with females having a higher intake median (IQR) 108 mg (49–192) compared to males 75 mg (19–132). There was no significant difference (*p* = 0.84) in the median (IQR) for caffeine (mg) between high 92 mg (33–187) and low 102 mg (26–148) SES groups.

Table 2 shows that more energy (*p* < 0.001) and % energy from protein (*p* = 0.04) was reported for males compared with females. There were no significant differences between genders for dietary fibre or % energy from total and saturated fat, carbohydrate or alcohol. For those participants consuming alcohol the mean intake for females was 19 g (28) and males 27 g (26). A significant difference (*p* = 0.01) was observed for dietary fibre (g), with participants from higher SES groups having a higher median (IQR) intake 24 g (19–31) compared to lower SES groups 19 g (14–28).

### 3.3. Intake of Fruit, Vegetables and Dairy/or Alternatives by Serves

Table 3 shows the median intake of fruit, vegetables and dairy and/or alternatives per day by gender, compared with the recommended serves for each food group [10]. Both males and females did not meet recommendations for fruit, vegetables or dairy and/or alternatives with no significant difference found between genders for all food groups. When comparing SES groups there was no significant difference observed for fruit, vegetables or dairy and/or alternatives.

### 3.4. Contribution of Different Foods to Calcium in the Diet

Table 4 shows that milk products and dishes provided the highest proportion of calcium for both females 30% and males 35%. Within the major group, the sub-major group of dairy milk provided (7%, 15%), yoghurt (4%, 3%), cheese (12%, 14%), frozen milk products (2%, 1%) and flavoured milks and milkshakes (5%, 2%) for females and males respectively. Milk products and dishes provided the highest calcium (mg) per portion 204 mg (212).

Cereal-based products and dishes were the second largest contributors of calcium intake after milk products and dishes for both females 16% and males 19%. The sub-major group, cereal-based mixed dishes contributed (13%, 17%) for females and males respectively, which included pizza (7%, 8%), burgers (1%, 2%) and savoury pasta/noodle dishes (3%, 3%). Calcium (mg), per portion was high for pizza, 460 mg (453), due to the mean portion size of 255 g.

Non-alcoholic beverages also made a contribution to calcium intake for both females 13% and males 10%. This group included items prepared with or without milk such as tea and coffee. The sub-major group, coffee and coffee substitutes contributed 7% for females and 6% for males, calcium (mg), per portion 100 mg (110).

Dairy and meat substitutes made a small contribution to calcium intake (mg) for both females 5% and males 2%. The sub-major group, meat substitutes (includes tofu, soy bean curd skins, tempeh and vegetarian sausages) contributed high amounts of calcium (mg) per portion 262 mg (173) and per 100g 368 mg (149), but these products were consumed infrequently for both females (*n* = 23) and males (*n* = 8).

The major group with the highest calcium (mg) per 100 g was special dietary foods (included meal replacement, sport and protein beverages, bars and powders) 505 mg (78), but made a minor contribution as average portion consumed was only 20 g.

## 4. Discussion

The present study demonstrates that many of these young adult participants are consuming inadequate amounts of desirable foods and nutrients in combination with nutrients that are deleterious for optimal bone health. Low intakes of fruit, vegetables and dairy products, as well as high sodium and alcohol, were common dietary behaviors for this sample of young adults putting them at risk for future osteoporosis. Given that adolescence and emerging adulthood is a time for PBM attainment, these dietary behaviors during this vulnerable period should be addressed.

There is strong evidence suggesting that calcium is an important nutrient for maximization of BMD and strength during periods of growth and development, with deficiency linked to osteoporosis [7]. The NHS conducted in 2011–2012 showed that 71% of females and 44% of males had intakes below the EAR [28]. This study highlights that calcium remains a nutrient of concern with 53% of females and 48% of males of this present study having intakes below the EAR which is considered at risk of inadequate intake. Further, both males and females in this sample had intakes well below the recommended average daily number of serves for dairy and/or alternatives. While socio-economic factors can influence lifestyle risk factors, this study showed no significant differences between individuals in the highest or lowest SES groups for calcium intake or consumption of dairy and/or alternatives serves.

Despite the rise in popularity of dairy and meat substitutes and other calcium alternative sources, milk products and dishes made the largest contribution to dietary calcium in the diets of these young adults. This study also showed a substantial amount of calcium came from non-alcoholic beverages (tea and coffee) and potentially less healthy sources including cereal-based dishes (pizza and burgers). However, it is the dairy component of these foods that is largely responsible. Increasing evidence suggests that fruit and vegetables have a positive influence on bone health as they contain micronutrients and alkaline performing properties involved in bone mineralization [11,12]. While no difference was observed for fruit and vegetable serves for SES, results from this study show that the mean daily intake of fruit and vegetables for females (0.6, 3.4 servings) and males (0.4, 2.8 servings) were well below recommendations. This is in line with previous research where fruit and vegetable intake was suboptimal, particularly for those aged 18 to 24 years [29]. Further, future educational interventions to dispel common myths around popular diets that omit major food groups and lead to inadequate calcium intakes with significant public health implications are required. A cross-sectional study showed that 11.8% of Australian adults aged 18 and over were avoiding dairy products due to physical symptoms without any formal medical advice [30].

Given the current food environment, high salt diets are a global concern as it is ubiquitous in the food supply. The majority of sodium in the diet comes from packaged processed food and food prepared outside of the home environment such as restaurants and fast food outlets [31]. Given young adults are consuming fewer home prepared meals and more meals prepared outside the home, it is not surprising that sodium usually exceeds recommendations [32]. Alcohol also has deleterious effects on bone and a recent study in adolescents and young adult women found an association between frequent heavy episodic drinking and reduced BMD [33]. This present study showed that alcohol contributed 5% of energy for females and 7% for male consumers. Although the contribution of energy from alcohol has decreased over time for young adults [34], heavy episodic drinking is more common compared to any other age group. In 2016, 42% of young adults aged 18 to 24 exceeded the single occasion risk guidelines by consuming more than four standard drinks on any one occasion [35]. For caffeine, the upper limit for healthy adults aged 19 and above is 400 mg [36]. The data suggest that caffeine intake in the range consumed by this sample is not an important risk factor for osteoporosis as the mean daily caffeine content consumed was ~ one cup of coffee (100 mg) for females. Further, studies have shown that the negative effect on caffeine on calcium absorption is minimal and can be offset by adding 1–2 tablespoons of milk [37], which confirms that young adults are still benefiting from calcium in beverages such as a cappuccino.

Public health messages reinforcing recommendations promoting bone health remain important for this age group. A qualitative study highlighted that young adults recognized the importance of dietary calcium for children and older adults, but not for their present age category [38]. The focus groups revealed that the mode of communication was important, and that nutrition related information would be better received if the information was delivered by trusted health professionals compared to other avenues such as social media [38]. This emphasizes the need to address and reinforce messages regarding adequate intake of essential nutrients.

The current study is not without some limitations. The 24 h dietary recall is prone to measurement error by inaccurate recall or food estimation however, this study found lower levels of low energy reporting compared to the NHS [22]. As the focus of the research was diet, supplements were not included which may change the percentage of individuals meeting the EAR for calcium. Vitamin D was not estimated in the present study as it was not in the Australian food composition database. However, sun exposure has been shown to be the main determinant of vitamin D status in Australia [39] and only 31% of those aged between 18–34 years in Australia have been reported to be vitamin D deficient [40]. The sample may not be generalizable to all young adults in NSW, Australia as more had post-school qualifications, more were from higher SES areas, less were living in major cities and normal weight was over-represented. Finally, no direct bone density measurements were taken for critical evaluation of BMD.

## 5. Conclusions

In conclusion, all young adults are advised to follow a healthy diet to maximize and maintain bone health. This study illustrates that the diets of young adults in this sub-sample was poor. As young adulthood is the final chance for dietary and lifestyle manipulation before PBM is achieved, identifying and correcting dietary behaviors that place young adults at risk for future osteoporosis should be addressed.

## Figures and Tables

**Table 1:** Sample characteristics.

Participant Characteristics		*n* (%)
Gender	Male	87 (46)
	Female	102 (54)
Age (years)	18–24	105 (56)
	25–30	84 (44)
Socio-economic Status ^1^	High (>50th percentile)	121 (65)
	Low (≤50th percentile)	64 (35)
Highest Education Attained	Secondary school or less ^2^	66 (35)
	University degree, trade or diploma qualification	123 (65)
Country Born	Australia	127 (67)
	Other/prefer not to say	62 (33)
Geographic Location ^3^	Sydney	125 (66)
	Outer Sydney	48 (26)
	Regional	15 (8)
BMI (kg/m^2^)	Under and normal weight (≤24.99)	120 (63)
	Overweight (25–29.99)	47 (25)
	Obese (≥30)	22 (12)
Misreporting ^4^	Low energy reporters	28 (15)
	Plausible reporters	155 (82)
	Over-reporters	6 (3)

^1^ Socio-economic status (SES) assessed using residential postcode to assign the index of relative socio-economic advantage and disadvantage centile employed within Australia, lowest five deciles = lower, highest five deciles = higher [19]. Four participant’s postcodes did not have an assigned decile. ^2^ Includes participants studying. ^3^ Assessed by the Accessibility Remoteness Index of Australia [20]. One participant with missing data. ^4^ Low energy reporters are participants with an energy intake: basal metabolic rate of ≤1.00, over-reporters ≥2.4 [23].

**Table 2:** Differences in energy, dietary fibre and macronutrients (g and % energy) between genders.

Energy and Nutrient Densities	Females (*n* = 102) Mean (SD)	Males (*n* = 87) Mean (SD)	*p* ^1^
Total Energy with Dietary Fibre, kJ	9298 (2467)	10712 (3217)	<0.001
Protein (g)	97 (31)	119 (43)	<0.001
Protein, % Energy	18 (4)	19 (5)	0.04
Total Fat (g)	93 (30)	104 (37)	0.03
Total fat, % Energy	37 (7)	36 (6)	0.28
Total Saturated Fat (g)	34 (14)	37 (16)	0.12
Total Saturated Fat, % Energy	13 (4)	13 (3)	0.20
Carbohydrate (g),	223 (74)	253 (88)	0.01
Carbohydrate, % Energy	41 (9)	40 (6)	0.28
Alcohol (g) ^2^,	19 (28)	27 (26)	0.15
Alcohol, % Energy	5 (6)	7 (7)	0.12
Dietary Fibre (g)	24 (10)	24 (9)	0.54

^1^ Independent sample *t*-test, *p* < 0.05 considered significant. ^2^ Alcohol calculation based on consumers only; 45 males and 54 females.

**Table 3:** Average number of serves from fruit, vegetables and dairy and/or alternatives food group by gender and compared to the recommended number of serves from the Australian Guide to Healthy Eating.

Food Group	Females (*n* = 102) Median (IQR ^1^)	Males (*n* = 87) Median (IQR ^1^)	*p* ^2^
Recommended Number of Serves for Fruit ^3^	2	2	
Fruit, dried fruit and all fruit juice	0.9 (0.4–2.0)	0.7 (0.2–1.4)	0.07
Fruit, dried fruit excluding fruit juice > 125 mL	0.6 (0.2–1.1)	0.4 (0.1–0.9)	0.05
Recommended Number of Serves for Vegetables ^3^	5	6	
Vegetables including Fried Potatoes and Vegetable Juice	3.8 (1.9–5.6)	3.4 (2.2–5.0)	0.78
Vegetables including Juice < 125 mL, excluding Fried Potatoes	3.4 (1.7–5.3)	2.8 (1.8–4.2)	0.31
Vegetables including Juice < 125 mL, excluding all Potatoes	3.0 (1.3–4.7)	2.6 (1.4–4.1)	0.46
Recommended Number of Serves for Dairy and/or Alternatives ^3^	2.5	2.5	
Dairy and/or Alternatives	1.1 (0.7–1.8)	1.3 (0.8–2.0)	0.11

^1^ IQR = interquartile range (25th and 75th percentile). ^2^ Mann–Whitney U test. ^3^ Recommended number of serves from the Australian guide to healthy eating (AGHE) [10]. A serve of juice = 125 mL.

**Table 4:** Contribution of selected major, sub-major and minor food groups to calcium (mg), per portion and per 100 g and the proportion contribution of calcium (mg) by gender.

Food Groups ^1^		Calcium (mg), Mean (SD)		Females		Males	
Selected Major Selected Sub-Major Selected Minor	Mean Portion Size (g) ^2^	Per Portion	Per 100g	*n* ^3^	Proportion ^4^ %	*n* ^3^	Proportion ^4^ %
Milk products and dishes	127	204 (212)	324 (300)	447	30	376	35
Dairy milk (cow, sheep and goat)	202	222 (234)	117 (36)	117	7	134	15
Yoghurt	137	236 (143)	171 (26)	48	4	37	3
Cheese	32	211 (230)	672 (202)	164	12	146	14
Frozen milk products ^5^	95	88 (54)	93 (14)	52	2	26	1
Flavoured milks and milkshakes	418	336 (178)	79 (21)	33	5	18	2
Cereal-based products and dishes ^6^	162	114 (209)	65 (56)	436	16	365	19
Cereal-based mixed dishes	264	211 (281)	84 (70)	168	13	186	17
Pizza	255	460 (453)	175 (60)	44	7	40	8
Burgers	185	158 (80)	90 (36)	16	1	31	2
Savoury pasta/noodle dishes ^7^	402	168 (142)	45 (31)	47	3	45	3
Non-alcoholic beverages	314	77 (105)	24 (56)	566	13	397	10
Tea^8^	327	41 (93)	10 (22)	176	2	78	2
Coffee and coffee substitutes ^8,9^	278	100 (110)	34 (37)	195	7	131	6
Other beverage flavourings/prepared beverages ^8,10^	188	225 (200)	153 (175)	31	2	17	8
Cereals and cereal products	108	53 (70)	70 (91)	463	9	410	10
Regular breads, and bread rolls	70	58 (38)	82 (28)	201	4	173	5
Breakfast cereals, ready to eat	62	83 (120)	162 (201)	53	2	60	2
Vegetable products and dishes	82	23 (55)	34 (122)	875	9	650	5
Dairy and meat substitutes	144	183 (183)	1712 (158)	79	5	24	2
Dairy milk substitutes, unflavoured ^11^	157	104 (114)	77 (36)	48	2	14	1
Meat substitutes ^12^	70	262 (173)	368 (149)	23	2	8	1
Meat, poultry and game products and dishes	158	25 (32)	15 (11)	362	3	294	4
Chocolate and chocolate-based confectionery	35	57 (84)	164 (75)	91	2	44	1
Fruit products and dishes	111	15 (20)	15 (16)	312	2	180	1
Fish and seafood products and dishes	122	63 (123)	50 (78)	74	1	63	2
Egg products and dishes	87	49 (42)	53 (19)	83	1	66	2
Special dietary foods ^8,13^	20	100 (67)	505 (78)	27	1	37	2

^1^ Foods were classified according to the Australian National Health Survey (NHS): User’s Guide, 2011–2013 Data item list [22]. This table only shows major, sub-major and minor food groups contributing ≥ 1.5% of total intake for either gender, ^2^ Amount consumed per eating occasion, ^3^ Number of items reported for each gender per category, ^4^ Percent of foods reported in each major food category, e.g., non-alcoholic beverages comprised 13% for females, ^5^ Includes ice cream, frozen yoghurt, gelato, sorbet and sundaes, ^6^ dishes refers to any food contained within a dish that is predominantly the food group mentioned (e.g., cereal-based products includes mixed dishes where cereal is the major ingredient such as pizza, burgers, pies, quiche, cookies, cakes, muffins and pastries), ^7^ Includes mixed pasta dishes where pasta is the main ingredient, e.g., all components of a spaghetti bolognaise including the pasta, sauce and cheese were included in this group, ^8^ Includes items prepared with or without milk, ^9^ Coffee substitutes include coffee prepared from coffee mixes, ^10^ Beverage flavouring include items such as Milo or Nesquik, ^11^ Includes soy or nut based products or beverages, ^12^ Includes tofu, soy bean curd skins, tempeh and vegetarian sausages, ^13^ Includes meal replacement, sports/protein powders.
